# The effects of earthquakes on children with autism: teachers’ post-earthquake observations and experiences

**DOI:** 10.3389/fpsyt.2026.1849866

**Published:** 2026-08-20

**Authors:** Mehmet Akif Kay, Osman Tayyar Çelik, Muhammed Abdulbaki Karaca, Özlem Aslan-Bağcı

**Affiliations:** 1 Department of Child Care and Youth Services Vocational School of Social Sciences, Batman University, Batman, Türkiye; 2 Department of Child Development, Faculty of Health Sciences, Inonu University, Malatya, Türkiye; 3 Department of Special Education, Faculty of Education, Inonu University, Malatya, Türkiye; 4 Department of Special Education, Faculty of Education, Sakarya University, Sakarya, Türkiye

**Keywords:** autism spectrum disorder, earthquake, special education, teacher perspectives, thematic analysis

## Abstract

This study aims to examine the behavioral, emotional, and academic changes observed in children diagnosed with autism spectrum disorder (ASD) following the February 6, 2023 Kahramanmaraş-centered earthquakes, based on the perspectives of special education teachers. The study was designed as qualitative research and conducted with 22 special education teachers working in the provinces most severely affected by the earthquake, namely Kahramanmaraş, Malatya, Hatay, and Adıyaman selected through criterion plus snowball sampling. Data were collected through semi-structured interviews and analyzed using thematic analysis, following an inductive approach. The findings were organized under three main themes: (1) the multidimensional impacts of the earthquake on children with ASD, (2) the restrictive effects of educational and living environments, and (3) decline in academic skills and learning difficulties. Teachers reported perceived increases in problem behaviors, regressions in social interaction and communication, emotional exhaustion, and losses in psychomotor and self-care skills that they associated with the earthquake, alongside co-occurring circumstances such as displacement, socioeconomic hardship, and interrupted access to education. In addition, inadequate educational conditions and environmental factors that continuously triggered traumatic memories were described by teachers as significant barriers to recovery. Based on these teacher perspectives, the results suggest a need for trauma-informed, comprehensive, and sustainable special education and psychosocial support services to support the post-disaster recovery and educational continuity of children with ASD.

## 1 Introduction

Natural disasters are events that profoundly disrupt the social, economic, and psychological balance of societies and radically affect the lives of individuals. Earthquakes, in particular, have been shown to engender long-lasting traumatic effects on individuals due to their sudden nature and devastating consequences (1, 2). Turkey, which is located on active fault lines, has experienced numerous devastating earthquakes throughout its history. Two major seismic events, centered in Kahramanmaraş on 6 February 2023, directly impacted eleven provinces, resulting in the demise of over 50,000 individuals, injuries to over 100,000, and the displacement of millions (3). This disaster has profoundly shaken the social structure, not only physically but also in its psychosocial and economic dimensions (4, 5). The consequences of disasters on children have been demonstrated to be more severe than on adults. It is well-documented that children are developmentally more sensitive to environmental and emotional changes. Consequently, they experience the uncertainty, fear and loss caused by disasters more intensely. It is particularly evident that children with special needs demonstrate an elevated degree of vulnerability during this period (6).

The developmental differences, communication limitations and sensory sensitivities serve to make the post-disaster recovery process significantly more complex (7). Children diagnosed with autism spectrum disorder (ASD) represent one of the most vulnerable demographics in this regard. This is attributable to the fact that such children are acutely sensitive to unexpected changes, may exhibit severe behavioral reactions if their routines are disrupted, and may have difficulty understanding environmental changes (8, 9).

In this context, the study can be evaluated within the framework of Bronfenbrenner’s (10) ecological systems approach and post-traumatic adaptation models (11, 12). This theoretical framework, by considering the individual’s development in interaction with environmental systems, allows for a multi-layered explanation of the effects of traumatic events, such as disasters, on children’s cognitive, emotional, and social areas. Concurrently, post-traumatic adaptation models emphasize that individuals’ responses to stress and crisis situations are shaped not only by individual factors but also by social support and environmental conditions. In this regard, the findings of Dimachkie Nunnally et al. (13) also reveal a strong relationship between environmental systems and psychosocial resilience. Research indicates that the psychological adaptation of children diagnosed with ASD and their parents to disaster or stressful situations is contingent not only on individual factors but also on social support, family resilience, and access to environmental resources. The present study thus seeks to address the adaptation difficulties experienced by children diagnosed with ASD in the post-disaster period. This is achieved by considering not only individual characteristics, but also the functioning of environmental systems and support mechanisms. It can be argued that the combination of the unique challenges faced by children with ASD and the inadequate consideration given to individuals with special needs in disaster management results in heightened societal vulnerability. Indeed, as asserted by the United Nations Office for Disaster Risk Reduction (14), it has been determined that 85% of individuals with special needs are not included in disaster risk reduction plans, 71% do not possess a personal disaster preparedness plan, and only 20% can be evacuated in an emergency. This situation demonstrates that individuals with special needs encounter challenges in disaster management processes (15, 16).

During disasters, individuals with special needs are at higher risk, both physically and psychosocially, due to factors such as poverty, lack of access to information, and physical disabilities (17, 18). In the aftermath of the 2023 earthquakes in Turkey, which had their epicenter in Kahramanmaraş, it was observed that individuals with special needs experienced significant difficulties in accessing housing, hygiene, transportation, communication, and health services (19). Children diagnosed with ASD have experienced significant challenges when adapting to crowded and noisy environments; these children have shown behavioral and emotional disturbances due to reasons such as disruption of their regular routines, lack of access to medication, and inability to access special education services (20). Furthermore, in individuals diagnosed with autism, social isolation, aggression such as breaking/throwing objects and harming others, increased repetitive behaviors, and an increased need for physical closeness to parents (separation anxiety) are highlighted (21). These findings are consistent with the view that autistic children may be particularly vulnerable to the challenges associated with traumatic experiences. In situations of crisis, such as earthquakes, teachers assume a dual role, functioning not only as conduits of educational processes but also as providers of psychosocial support. Special education teachers are critical actors who can observe and understand the emotional responses of children with ASD, help them restructure their routines, and create safe environments (22). A number of qualitative studies conducted in Turkey subsequent to 2023 have indicated that educators have observed substantial alterations in the play behaviors, communication styles and overall motivation of children following the earthquake. In this context, a systematic examination of teachers’ opinions will make significant contributions to the development of post-disaster special education policies and the structuring of psychosocial support programs to be implemented in the field (23).

Natural disasters prevent many children from continuing their education, create difficulties in accessing educational materials, and ultimately lead to their deprivation of the right to education (24). While all children are affected by disasters, those with ASD may be particularly vulnerable (25). In the education of children with ASD, families, systematic instruction, an open/structured learning environment, special curriculum content, a functional approach to challenging behaviors, and family participation are of great importance (26). Disasters with devastating effects, such as earthquakes, often complicate and render this process inaccessible. This situation highlights alternative educational models, such as post-disaster online education, for children with ASD. In this type of education, the inability of teachers to effectively convey certain commands (e.g., physical spatial) remotely creates limitations in children’s responsiveness (27).

The current literature has mostly examined the impact of disasters on individuals with ASD based on parental perspectives (20, 21, 28). Furthermore, these studies have touched upon the emotional and behavioral problems experienced by children with ASD after disasters, albeit in a limited way. However, it should be noted that studies based on teachers’ observations are extremely limited. Teachers are in a unique position to understand the developmental impacts of disasters because they can directly observe children’s daily behavioral changes, differences in communication styles, and regressions in learning processes. Therefore, research based on the teacher perspective is critical for the restructuring of special education services after disasters (23). Special education teachers provide an important perspective on the post-disaster experiences of children with ASD because they observe students in academic, social, communication, and adaptive functioning contexts within educational settings. Through their ongoing interactions with students, teachers can identify developmental changes and skill regressions over time. Therefore, teacher-based observations may complement parent-reported findings by offering additional insights into children’s educational and developmental experiences following disasters.

The aim of this research is to identify any potential losses in behavioral and academic skills acquired prior to the earthquake in children with ASD. This will be based on the opinions of special education teachers working in the regions affected by the February 6, 2023, Kahramanmaraş-centered earthquakes. It also aims to examine the emotional and behavioral problems that may have contributed to these losses after the earthquake, through teacher observations. Additionally, the research aims to evaluate the difficulties encountered in post-disaster special education practices and the adequacy of existing support mechanisms, and to develop recommendations for strengthening the educational recovery and readjustment processes of children with ASD.

## 2 Materials and methods

### 2.1 Research model

This study, which evaluates the effects of earthquakes on autistic children based on teachers’ post-earthquake perspectives, utilizes a qualitative research design. Qualitative research approaches aim to gain a comprehensive understanding of phenomena, focusing on individuals’ experiences of social phenomena within their environment and their in-depth examination of their meaning (29, 30).

### 2.2 Working group

The study group consisted of 22 special education teachers working in the provinces of Kahramanmaraş, Malatya, Hatay, and Adıyaman, which were most severely affected by the February 6, 2023 Kahramanmaraş-centered earthquakes. The criterion sampling technique, a type of purposeful sampling method, was used to select participants. Criterion sampling involves including individuals who meet predetermined criteria relevant to the study’s objective (31). Inclusion criteria in this context are as follows: (1) working in one of the provinces that was severely impacted by the February 6, 2023 Kahramanmaraş-centered earthquakes (Kahramanmaraş, Malatya, Hatay, and Adıyaman), (2) actively working with students diagnosed with ASD prior to the earthquake, (3) continuing in their duties after the earthquake and being able to observe their students diagnosed with ASD again. In addition, snowball sampling was used to reach the participants. Snowball sampling is a method that is often used to reach populations that are difficult to access (32). Information about the teachers participating in the study is given in **Table 1**. Examining the age distribution of the participants, it is seen that the majority of participants (54.5%) are in the 25–34 age range, followed by 31.8% in the 35–44 age range and 13.7% in the 45–54 age range. Regarding the distribution of the provinces where the participants work, it is seen that the vast majority of teachers (36.3%) work in Malatya, 22.7% in Kahramanmaraş, 22.7% in Hatay, and 18.1% in Adıyaman. Looking at the distribution regarding teaching experience, the highest percentage (45.5%) consists of teachers with 0–5 years of experience, followed by 22.7% with 6–10 years, 18.1% with 16 years and over, and 13.7% with 11–15 years of experience. In terms of institutional context, 10 teachers (45.5%) were employed in first-stage special education schools, whereas 12 teachers (54.5%) were employed in second-stage special education schools. According to the Special Education Services Regulation (33), special education practice centers established for individuals with moderate or severe intellectual disabilities and individuals with autism are organized into educational stages. The first four years, covering Grades 1, 2, 3, and 4, are referred to as the first stage, while the second four years, covering Grades 5, 6, 7, and 8, are referred to as the second stage. The ages of the children with ASD taught by the participating teachers ranged from 7 to 14 years. All students with ASD included in the teachers’ observations were reported to have moderate-level ASD.

**Table 1:** Demographic and occupational information of the study group.

Variable	Category	f	%
Age Range	Ages 25-34	12	54.5
	Ages 35-44	7	31.8
	Ages 45-54	3	13.7
The period he/she worked as a teacher	0–5 years	10	45.5
	6–10 years	5	22.7
	11–15 years	3	13.7
	16 year and older	4	18.1
Province of Residence	Malatya	8	36.3
	Kahramanmaraş	5	22.7
	Hatay	5	22.7
	Adıyaman	4	18.1
Type of Institution	First-stage special education school	10	45.5
	Second-stage special education school	12	54.5
Total Number of Participants		22	100

### 2.3 Data collection tool

A semi-structured interview form developed by the researchers was used as a data collection tool in the study. The interview form aims to holistically reveal teachers’ observations regarding the education, social interaction, behavioral adjustment, and emotional responses of autistic children in the post-earthquake period. During the development process, a comprehensive literature review was conducted on the education of individuals with special needs after a disaster, post-traumatic development, and teacher experiences. Following this review, a draft form consisting of seven questions was created. While preparing the questions, care was taken to ensure that they served the research purpose, were clear and understandable, were single-layered and interrelated, non-judgmental, and complied with ethical principles (34, 35). The draft form was sent to two experts experienced in qualitative research on related topics, and they were asked to review the validity of the questions. Following the feedback, the number of questions was reduced to six, and simplified to improve their clarity. Additionally, a pilot interview was conducted with a teacher to evaluate the effectiveness of the questions and the interview duration. After the interview, it was decided that the questions were sufficient, clear, and understandable, and the form was finalized. The interview form, consisting of two sections, includes a personal information form containing demographic information of the participants and six semi-structured questions regarding their observations after the earthquake.

### 2.4 Data collection

The research data was collected after approval by the Batman University Ethics Committee, decision number 2024/06-27. Data were collected between July 1 and August 5, 2024, approximately 17–18 months after the February 6, 2023 earthquakes. This timing was related to the post-earthquake displacement of many children with ASD and their families to other provinces, and their gradual return after living and educational conditions had been reorganized. In line with the aim of the study, the interviews focused on children with whom the teachers had worked before the earthquake and who returned to their educational settings after the earthquake. Snowball sampling was used to reach participants. In this context, special education teachers on the social networks of researchers specializing in special education were contacted and informed about the purpose of the research. A pool of potential participants was created from the information obtained from these teachers. The participant list was expanded with teachers contacted from this pool. Participants were given detailed explanations of the research’s purpose, scope, and confidentiality principles; it was stated that participation was entirely voluntary. Recruitment continued until the data provided sufficient depth and information richness to address the research aim.

Prior to the interviews, participants were contacted by phone, and the dates, times, and locations of the interviews were determined according to the participants’ preferences. The interviews were conducted in environments and on platforms deemed suitable by the teachers. While face-to-face interviews were conducted with eight participants located in Malatya Province, interviews with participants in other provinces were conducted via telephone calls and online meetings. Written informed consent was obtained from participants who took part in face-to-face interviews, while verbal informed consent was obtained from participants who were interviewed by telephone or online. Each interview lasted approximately 40 to 50 minutes. With the participants’ consent, all interviews were recorded.

### 2.5 Data analysis

Before data analysis, audio recordings were transcribed word for word and transferred to a computer. After the accuracy of the transcripts was checked by MAK, the transcripts were sent to the participants to confirm their accuracy. In data analysis, the six-stage inductive approach proposed by Braun and Clarke (36) for thematic analysis was adopted. This thematic analysis approach offers researchers the opportunity to flexibly explore and interpret meanings without being bound by any epistemological or theoretical perspective (37). In the second stage, meaningful statements reflecting the teachers’ post-earthquake observations and experiences were identified and labeled with initial codes. The coding process was conducted manually, with Microsoft Excel used to organize the codes and themes. To ensure coding consistency, two researchers (MAK and OTC) independently coded a section of the transcripts and subsequently compared their coding decisions. Differences regarding code names, code boundaries, and the placement of codes under potential themes were discussed in researcher meetings and resolved through consensus. At the end of this process, the list of codes was reviewed to establish a common coding framework, which was then utilized in the analysis of the remaining transcripts. Throughout the analysis, the researchers referred back to the original transcripts to verify that the codes and themes accurately reflected the participants’ statements. In the third stage, during joint meetings attended by all researchers, similar codes were grouped together to identify potential sub-themes and themes. In the fourth stage, the themes and codes were compared with the data set to check for consistency. In the fifth stage, the scope, boundaries and conceptual content of the themes were reviewed and named in line with the research objective. In the final stage, the findings were reported, supported directly by participant quotations.

To ensure reflexivity, the researchers conducted regular self-assessments throughout the analysis process regarding how their professional backgrounds and experiences in special education, child development and qualitative research might influence the data interpretation process. To prevent individual interpretations from becoming dominant, coding decisions and the theme development process were discussed within the team, and peer review was sought from a qualitative research expert not directly involved in the study. An audit trail comprising transcripts, initial codes, theme development notes and researcher notes was maintained throughout the process.

### 2.6 Trustworthiness

A series of measures were taken to ensure the validity and reliability of the research (38). In this context, interview questions were meticulously prepared and submitted for expert opinion. To increase credibility throughout the research process, researcher triangulation was applied at all stages. Researcher triangulation is a method where more than one researcher participates in the data collection, analysis, and interpretation phases (39). To support participants’ rights, participants were given the assurance that they could withdraw from the study at any time (40). Furthermore, sending transcripts to participants for verification contributed to the credibility of the research. In addition, by obtaining support from an expert who was not directly involved in the study throughout all stages, we ensured the inclusion of different perspectives through peer review (38) and prevented the dominance of a single dominant interpretation.

## 3 Results

**Figure 1:**
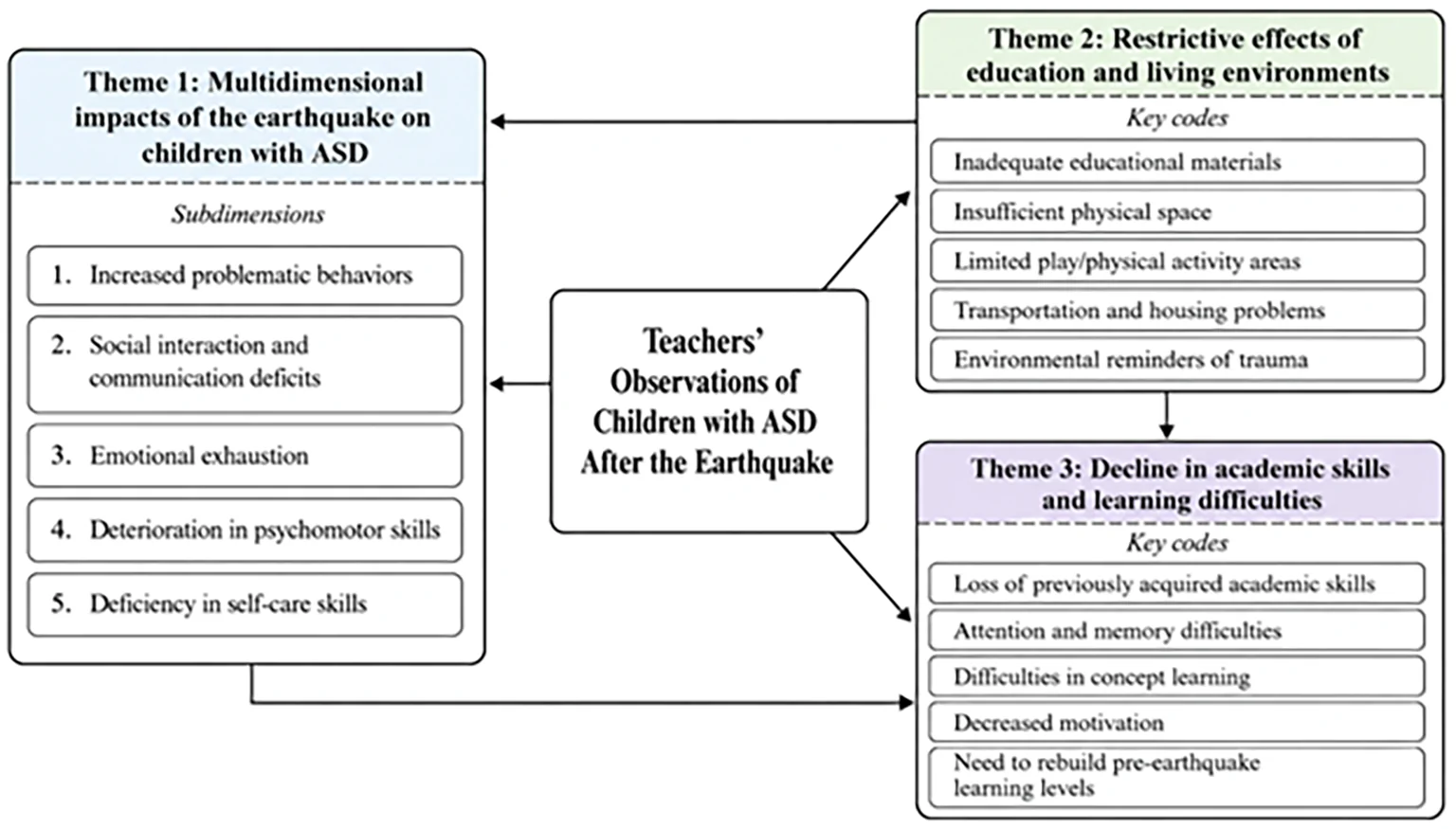
Themes, subthemes, and key codes.

As a result of the analysis of data collected from special education teachers to determine the effects of the 2023 Kahramanmaraş earthquake on children with ASD, three themes were identified. These themes, along with their associated sub-themes, are presented in **Figure 1**. These are; (1) the multidimensional effects of the earthquake on children with ASD, (2) the restrictive effect of education and living environments and (3) decline in academic skills and learning difficulties.

### 3.1 Theme 1: the multidimensional impacts of the earthquake on children with ASD

Special education teachers have reported numerous observations regarding the impairments and problems observed in various developmental areas of students with ASD following the earthquake. Under this general theme, five sub-themes were identified encompassing the impact of the earthquake on different developmental areas of children with ASD. These are: increased problematic behaviors, delayed social interaction and communication, emotional exhaustion, regression in psychomotor skills, and inadequate self-care skills.

#### 3.1.1 Increased problematic behaviors

Teachers frequently stated that problem behaviors increased after the earthquake. These behaviors included crying spells, temper tantrums, self-harming behaviors, panic responses, and intense fear reactions. However, these behaviors should not be interpreted only as general behavioral problems. Teachers’ accounts suggest that many of these reactions were closely related to trauma reactivity and sensory sensitivity. For example, one teacher stated:

“Some of my students panic and jump up and head towards the door when the desk shakes while they are using an eraser. I can calm them down with difficulty.” (Participant 1)

This statement shows that even a minor physical stimulus, such as the shaking of a desk, could function as a trauma reminder for children with ASD. The children’s sudden movement toward the door may indicate that the sensory experience of shaking reactivated the earthquake experience and triggered an intense fear response. From this perspective, the increase in problem behaviors was not simply a direct consequence of the earthquake itself, but also a response to post-disaster sensory cues that resembled the traumatic event.

Teachers also reported that previously controlled behaviors re-emerged or intensified after the earthquake. One teacher noted that behaviors that had been reduced before the earthquake became more visible again, while another teacher stated that one child began to put objects into their mouth constantly. These examples suggest that the disruption of routines and loss of predictable environments weakened children’s behavioral regulation. For children with ASD, predictable daily patterns, familiar classrooms, and consistent adult responses are important regulatory supports. When these supports were disrupted after the earthquake, children may have experienced greater anxiety, uncertainty, and sensory overload. Therefore, behaviors such as crying, panic, self-harm, oral sensory seeking, or sudden escape responses can be understood as attempts to cope with overwhelming emotional and sensory demands.

#### 3.1.2 Social interaction and communication deficit

Teachers also observed regression in children’s social interaction and communication. Children who had previously been more socially active became more withdrawn, less willing to communicate, and more likely to remain alone. One teacher described this change as follows:

“Perhaps due to the fear they experienced, the children, who used to be active and lively, have become withdrawn and unwilling to communicate.” (Participant 1)

This statement suggests that social withdrawal was not merely a continuation of ASD-related social difficulty, but may have been intensified by post-traumatic fear and emotional insecurity. Another teacher shared that she tries to focus on group training sessions to overcome problems related to social interaction, and that she tries to initiate communication at every opportunity.

#### 3.1.3 Emotional exhaustion

Teachers described many children with ASD as emotionally exhausted after the earthquake. They reported that children were less energetic, less willing to participate in activities, more easily distressed, and more likely to cry in response to minor difficulties. One teacher expressed this as follows:

“I’m having difficulty engaging the students in various activities. They seem to have lost their energy. They’re even reluctant to participate in fun activities.” (Participant 5)

This statement indicates that the earthquake affected children’s motivation and emotional availability for learning and play. The reluctance to participate even in enjoyable activities suggests that children were not only behaviorally affected but also emotionally depleted. Another teacher emphasized the role of family separation and insufficient family support:

“Some children are having difficulty concentrating on their lessons because they have been separated from their families during this period. They react by crying at the slightest problem.” (Participant 3)

This statement highlights family support as a protective factor. Teachers emphasized that families are a vital resource for children with ASD in terms of emotional security, the continuity of routines, the interpretation of stressful events, and self-regulation.

#### 3.1.4 Deterioration in psychomotor skills

Teachers reported stagnation and regression in psychomotor skills, including fine motor skills, gross motor skills, hand-eye coordination, and previously acquired physical abilities. These regressions were particularly visible in children whose therapeutic or educational routines had been interrupted. One teacher stated:

“I had made significant progress with a student of mine who was receiving physical therapy before the earthquake. However, all of that progress was wasted after the earthquake.” (Participant 9)

These views indicate that children with ASD may experience regressions in psychomotor skills and even lose many of their acquired skills if they do not receive adequate support after traumatic events such as earthquakes. Another teacher described difficulties in fine motor coordination:

“We are particularly seeing problems with hand-eye coordination. As a result, they seem to have lost their ability to hold a pen.” (Participant 10)

This observation also has implications for learning. Fine motor skills such as holding a pencil require attention, coordination, emotional readiness, and repeated practice. Post-disaster anxiety, hypervigilance, sensory overload, and reduced concentration may have indirectly affected children’s motor performance. In addition, another teacher stated:

“In psychomotor skills, as in other problems, students exhibit a high level of stiffness. Sudden running, sudden hand clapping, and repetitive behaviors can occur.” (Participant 4)

This quote suggests that psychomotor regression was accompanied by increased repetitive behaviors and bodily tension. Such behaviors may reflect sensory dysregulation and attempts to cope with overwhelming stimulation. Thus, the deterioration in psychomotor skills appears to be linked to the combined effects of interrupted routines, reduced therapeutic continuity, trauma reactivity, and sensory processing difficulties.

#### 3.1.5 Deficiency in self-care skills

Teachers frequently stated that children experienced regression in self-care skills such as toileting, hygiene, dressing, and eating. These regressions were more pronounced among children with greater support needs. One teacher described this situation as follows:

“Students with particularly severe self-care skills are experiencing a high level of incontinence problems in the post-earthquake period. Even things like wiping their noses with tissues have been almost completely forgotten.” (Participant 2)

This statement shows that self-care skills were not fully independent from context. For many children with ASD, self-care routines depend on repeated sequences, familiar physical environments, adult prompts, and predictable daily schedules. After the earthquake, many children had to live in tents, containers, or unfamiliar environments, and their daily routines were disrupted. In this context, regression in toileting or hygiene may reflect the loss of routine cues and environmental supports rather than a simple forgetting of skills. Another teacher similarly emphasized the impact of post-earthquake living conditions:

“The real experiences and the life they have to endure afterward have set our children back a notch in terms of self-care skills.” (Participant 3).

These opinions imply that students were in better shape in terms of self-care skills before the earthquake, but experienced a decline in these areas after the earthquake. Additionally, it appears that these declines are more pronounced in students with severe disabilities.

### 3.2 Theme 2: the restrictive effects of education and living environments

The damage and negative environmental impacts caused by the earthquake have also affected students with ASD and their educational processes. Within this context, teachers have highlighted the lack of educational materials, insufficient physical space, lack of areas for children’s physical activity (parks, playgrounds, etc.), the presence of factors in the environment that remind them of the earthquake (ruined buildings, cracks in houses, etc.), transportation problems, and housing problems under this theme. Some of these environmental challenges remind children of their traumatic experiences, while others involve inadequate conditions for education. Additionally, teachers emphasized the limited availability of resources to meet children’s social needs. One teacher emphasized the effect of destruction and displacement on children’s social lives:

“They are also experiencing difficulties socializing in the region due to the destruction and the displacement of people.” (Participant 7)

This statement indicates that the earthquake weakened the social ecology surrounding children with ASD. The destruction of familiar places and the displacement of people reduced children’s opportunities for social interaction. For children with ASD, who often need structured and familiar social contexts, this loss may have deepened social withdrawal and reduced opportunities for practicing communication and adaptive behavior. Another teacher pointed to access problems in special education:

“Students who were unable to receive special education support due to transportation issues in the places they went to because of the earthquake faced difficulties.” (Participant 6)

It is evident from teachers’ feedback that transport issues have not only led to absenteeism but have also disrupted the continuity of personalized education, routines and therapeutic support. For children with ASD, the loss of consistent educational input can increase uncertainty and make it difficult to maintain previously acquired skills. The lack of physical activity areas was also emphasized:

“The lack of places to engage in physical activity has made the students even more restless.” (Participant 3)

Physical activity can help children with ASD regulate their sensory input, reduce their restlessness and manage their anxiety. However, teachers have reported that environmental constraints contribute to behavioral difficulties.

As can be understood from the above excerpts, the earthquake damaged the areas where children socialize and the displacement of individuals from their immediate surroundings created a limited environment for students with ASD. In addition, families who moved to containers or other locations far from educational facilities experienced difficulties in accessing special education services. Furthermore, inadequate conditions for physical activity and a lack of materials in educational settings were also cited by teachers as factors that triggered the students’ anxiety.

### 3.3 Theme 3: decline in academic skills and learning difficulties

Teachers have reported that students with ASD who returned to school after the earthquake have forgotten previously acquired academic achievements (such as recognizing letters and counting). Furthermore, weakening of attention and memory processes, and difficulties in learning concepts are among the problems teachers face. Teachers also emphasized that they are primarily focused on bringing students back to their previous levels after the earthquake. In this context, the participant shared her disappointment regarding the academic decline of a student she had described as having reached an excellent level, while expressing her belief that the student could return to their former level. In addition, teachers frequently highlighted that students quickly forget newly acquired information and are unable to transfer it to long-term memory. In sharing these views, teachers referred to interruptions in the educational process, the emotional and psychological states of the students, environmental factors, and a lack of materials. Some direct quotes from the participants are as follows.

“The students’ academic performance is as if they received no education at all before *the earthquake; it’s like we’re starting from scratch, unfortunately.”* (Participant 1)

“Academic performance is at a level we absolutely do not want, and even our students with mild intellectual disabilities have significant attention deficit and concentration problems.” (Participant 6)

“Many of the gains we have achieved have to be worked on again, it has been forgotten.” (Participant 17)

As can be understood from the above excerpts, students with ASD experienced learning losses after the earthquake, and teachers focused on helping them regain these forgotten skills and behaviors. However, teachers emphasized that students with ASD had difficulties acquiring new knowledge and skills, including previously acquired knowledge and abilities, and that these students struggled to stay motivated.

## 4 Discussion

This study, which examines the effects of the 2023 Kahramanmaraş-centered earthquakes on children diagnosed with ASD, aims to fill an important gap in understanding the challenges faced by individuals with special needs after an earthquake. A salient feature of the study is that the data was obtained directly from special education teachers. A review of the extant literature reveals a paucity of research based on teacher opinions regarding autistic children exposed to the earthquake (23). This situation enhances the originality and potential contribution of the study; it also reveals the need for a reassessment of support mechanisms in the field of special education after the earthquake. The present study extends the existing literature by examining post-disaster experiences of children with ASD from the perspective of special education teachers. Unlike previous studies that have predominantly relied on parent reports, the current findings reflect observations obtained within educational settings and provide additional insight into children’s academic, social, communication, and adaptive functioning. Based on the data obtained, significant conclusions were reached under three main themes: emotional and behavioral problems experienced by children with ASD, the restrictive effects of educational and living environments on children, and academic skill regression and learning difficulties such as slowed cognitive processes. Findings can also be interpreted through Bronfenbrenner’s ecological systems theory and post-traumatic adaptation models. These frameworks suggest that children’s responses to traumatic events are shaped not only by individual characteristics but also by the interaction of multiple environmental systems (10, 41). Therefore, the effects observed among children with ASD following the earthquake should be understood within a broader ecological context involving family relationships, educational settings, support services, and disaster-related policies.

According to teachers’ observations, a significant increase in problematic behaviors was observed during the post-earthquake period. Patterns such as crying spells, temper tantrums, and self-harming behaviors indicate that the trauma has been intensely internalized. In addition, according to teachers’ opinions, there has been a decline in social interaction and communication, children have become more withdrawn, and have developed an aversion to social environments. The observed increase in social withdrawal may reflect more than a general post-traumatic response. For children with ASD, whose social communication and interaction skills are often dependent on predictable routines, familiar environments, and structured support, the abrupt disruption caused by the earthquake may have undermined important mechanisms that facilitate social engagement. Heightened uncertainty, environmental instability, and the loss of established social routines may have exacerbated pre-existing social communication difficulties, leading to greater social disengagement and withdrawal.

Emotionally, a general state of exhaustion and conditions that trigger anxiety disorders have emerged in most children. Teachers stated that this situation was even more severe in children who lacked sufficient family support or were forced to separate from their families in the education provided in the post-earthquake conditions. Furthermore, significant regressions occurred in psychomotor skills and self-care (daily living skills such as eating, dressing, and hygiene). These findings suggest that developmentally vulnerable groups may experience difficulties in maintaining life skills when their routines are disrupted during crisis periods. In their study, Özarslan et al. (42) concluded that children who experienced the earthquakes centered in Kahramanmaraş reported feelings of fear, anxiety, sadness and longing during and after the disaster. This vulnerability may be particularly pronounced among children with ASD, whose daily functioning often depends on highly structured routines, repeated practice, and environmental consistency. The displacement, changes in living conditions, and interruption of educational and therapeutic services following the earthquake may have disrupted these support mechanisms. Such disruptions may have contributed not only to emotional distress but also to the deterioration of previously acquired psychomotor and self-care skills.

The children also exhibited adverse changes in their sleep patterns, experienced difficulties in meeting their basic needs, and their private living spaces decreased, and their education processes were interrupted. These observations are consistent with the broader literature on disaster-affected individuals with special needs, who are often overlooked in disaster risk reduction planning and consequently face compounded obstacles in accessing basic services such as shelter, hygiene facilities, and medical supplies during and after disasters (15, 19). Healthcare workers have similarly reported heightened fear and anxiety among affected children (43). Areas that showed significant worsening during the pandemic in children with ASD were identified as irritability, social withdrawal, stereotypy, hyperactivity, inappropriate speech, self-harm, sleep disturbances, sleep duration, and sleep quality (44). Eroglu et al. (45) observed that children with ASD, who are particularly sensitive to routines, exhibited maladaptive behaviors after devastating earthquakes that changed their lives. Taken together, these difficulties appear to reflect the combined influence of earthquake-related stress, changing environmental conditions, disruptions in services, and instability in daily routines.

From an ecological systems perspective, these findings primarily reflect disruptions within the microsystem, which includes children’s immediate interactions with family members, teachers, and daily routines (46). For children with ASD, who often depend on predictable environments and stable interpersonal relationships, disturbances in these microsystem structures may have intensified emotional distress, communication difficulties, and behavioral challenges. Furthermore, post-traumatic adaptation models suggest that reduced emotional support and ongoing environmental instability can hinder recovery and increase vulnerability following traumatic events.

The restrictive impact of educational and living environments on children emerged as another important theme in the study. Following the earthquake, factors such as disruptions in access to educational environments for children with ASD, physically damaged schools, insufficient educational materials, unsuitable container living spaces for education, and the lack of open play areas negatively affected both the cognitive and social development of children. Furthermore, teachers reported that visual reminders such as ruined buildings and cracked walls appeared to reinforce traumatic memories and emotional distress. During this period, teachers stated that both the reconstruction of educational environments and the support to ensure the safety and comfort of students were insufficient. In this period of increased social isolation, the reduced opportunities for children with ASD to interact with their peers hindered developmentally critical social learning processes. These results imply that interruptions in special education and the trauma experienced may lead to clinical deterioration in children with ASD after the earthquake. Aslan’s (47) study indicated that school-aged students with ASD experienced severely limited or completely disrupted access to face-to-face education after the earthquake, depending on the level of destruction and impact in the affected region. According to the findings of this study, parents emphasized the importance of face-to-face education for their children and stated that regular school attendance was a critical element in meeting the developmental and social needs of their children. Özarslan et al. (42) stated that limited resources in the living spaces of earthquake-affected children, lack of private spaces, negative changes in the educational process, and uncertainties about the future led to decreased motivation and the emergence of various psychosocial problems. Among the factors associated with post-earthquake psychological distress, situations such as interruption of special education triggered earthquake trauma. In Hatay, one of the provinces where special education was interrupted for a longer period, more significant increases in autism symptoms, irritability, and hyperactivity levels were observed (45). In the study conducted by Şimşek et al. (21), it was reported that all parents experienced serious difficulties in their children’s access to education after the earthquake. Parents stated that this process had negative effects on their children’s developmental progress. In a similar study (44), it was found that although measures were taken to ensure the continuation of general education through distance education during the Covid-19 period, most individuals with ASD did not have sufficient access to special education. In conclusion, teachers reported that restrictions in educational and living environments during the post-earthquake period were associated with educational disruptions, developmental difficulties, and psychosocial challenges among children with ASD. Findings can be interpreted through the mesosystem and exosystem levels of Bronfenbrenner’s ecological framework. The weakening of interactions between families, schools, and special education providers reduced the continuity of support available to children with ASD (10, 46). In addition, barriers related to transportation, rehabilitation services, housing conditions, and educational resources represent exosystem factors that indirectly influenced children’s development and well-being. These findings highlight the importance of maintaining coordinated support systems following disasters.

According to the final theme that emerged from the study, children with ASD experienced cognitive regression and academic difficulties when they returned to their education after the earthquake. Teachers observed that children forgot previously acquired academic skills (e.g., letter recognition, counting) and showed weakening in attention and memory functions. Children who had difficulty learning concepts also struggled to transfer learned information to long-term memory. Teachers stated that they primarily tried to bring the students back to their pre-earthquake levels, but this effort required significant time and resources. In addition, the low motivation levels of the students further complicated the learning process. This situation suggests that psychological distress, educational disruption, and environmental instability may collectively influence learning capacity following traumatic events. Gibbs et al. (48) reported a significant decrease in academic achievement in reading and mathematics in children who experienced disasters; Gomez and Yoshikawa (49) reported that earthquake experiences had negative effects on language and pre-literacy skills in early childhood. Furthermore, Yılancıoğlu and Özbaran (50) stated that in the post-disaster period, children experienced loss of learned skills, difficulties in regaining these skills, and impairments in attention and memory processes. Similarly, a study conducted by Kay et al. (20) emphasized that the disruption of learning processes led to significant regressions in many previously acquired social, communicative, and cognitive skills in children with ASD. Academic regression should be understood not merely as an individual learning difficulty but as the cumulative result of disruptions occurring across ecological systems (46, 51). Reduced educational opportunities, interruptions in teacher–student interactions, weakened family support mechanisms, and limited access to specialized services collectively contributed to learning losses. Consistent with post-traumatic adaptation models, prolonged exposure to stress and instability may negatively affect attention, memory, and learning processes, thereby increasing the likelihood of academic regression. In addition to the psychological effects of the disaster, many children experienced prolonged displacement, changes in living conditions, and socioeconomic challenges that may have altered their access to educational resources and learning opportunities. These broader post-disaster conditions may also help explain the academic difficulties and skill regressions reported by teachers.

## 5 Conclusions and limitations

This study, based on teacher opinions, offers important insights into the emotional and behavioral problems experienced by children with ASD in the post-earthquake period, the restrictive effects of educational and living environments on these children, and the regressions in cognitive and academic skills. Evaluated based on the opinions of special education teachers, this study suggests that, in addition to the direct effects of trauma, inadequacies in educational and living environments may have further deepen these effects and appear to represent important factors associated with educational losses and reduced learning capacities. Given the retrospective, teacher-report nature of the data, these co-occurring circumstances cannot be fully disentangled from the direct effects of the earthquake itself, and should be interpreted as interrelated rather than independent factors. In this context, post-disaster support for children with ASD should extend beyond trauma-focused psychosocial interventions. The findings suggest that restoring educational continuity, ensuring uninterrupted access to special education and rehabilitation services, rebuilding structured learning environments, and re-establishing predictable daily routines are equally important for recovery. Teachers frequently emphasized that many children appeared to experience skill loss following prolonged disruptions in educational services and daily routines. For children with ASD, the restoration of familiar routines and stable educational environments may itself function as a protective and therapeutic intervention by reducing uncertainty, supporting emotional regulation, and facilitating the recovery of previously acquired skills. Furthermore, identifying potential risks that individuals with ASD may face before and after disasters and developing special intervention programs in advance is another crucial aspect.

When interpreted through Bronfenbrenner’s ecological systems framework, the findings suggest that the effects observed during the post-earthquake period may extend beyond individual trauma and appear to be reflected across multiple interconnected environmental systems. Disruptions within the microsystem (family and teacher relationships), mesosystem (school–family collaboration), exosystem (access to educational and support services), and macrosystem (disaster policies and social structures) —as well as broader circumstances such as displacement and socioeconomic hardship experienced by families—may have collectively contributed to the difficulties children with ASD experienced. Findings suggest that post-disaster interventions should be planned not only at the individual level but also across ecological systems to strengthen resilience, improve educational continuity, and support long-term recovery. When interpreting the results of this study, it is important to consider its limitations. Because the research is qualitative in nature and focuses on the experiences of a limited number of participants, the generalizability of the results to a wider population is limited. Therefore, future research could focus on existing variables in larger populations using quantitative methods regarding emotional and behavioral problems and educational skill regression in children with ASD after earthquakes. Furthermore, longitudinal studies could observe results over time. Another limitation concerns the timing of data collection. Since the interviews were conducted approximately 17–18 months after the earthquakes, children’s post-earthquake adjustment, access to support services, and changing living conditions during this interval may have shaped the clinical and educational picture described in the study. Another limitation of the study concerns the selection of participants. The research was conducted with participants from the provinces most severely affected by the earthquake. This may have limited the reflection of experiences in different contexts. Furthermore, the fact that the study group consisted solely of special education teachers working in specific provinces most severely affected by the earthquake limits the generalizability of the findings to other disaster-affected regions or different institutional contexts. The uneven distribution of participants’ levels of professional experience and the higher proportion of young teachers in the sample may also have affected the quality of the teachers’ observations. Therefore, it is recommended that future studies include teachers with varying levels of experience, different types of institutions, and children with ASD who have different support needs in a more balanced manner. Finally, the data were obtained from teacher interviews. Future research could focus on the effects of disasters on children with ASD by utilizing data diversity through interviews with parents and direct observations of children.

## Data Availability

The original contributions presented in the study are included in the article/**Supplementary Material**. Further inquiries can be directed to the corresponding author.
