# Supplementary material for: The effects of earthquakes on children with autism: teachers’ post-earthquake observations and experiences
Source: Front Psychiatry. 2026 Aug 20;17:1849866. doi: 10.3389/fpsyt.2026.1849866 (PMC13538887; doi:10.3389/fpsyt.2026.1849866)
Supplement: Supplementary file 1 [file Supplementaryfile1.docx]

Supplementary Material

# 1 Supplementary Figure

**Supplementary Figure 1.** Themes and sub-themes
